# Supplementary figures and images for: AMPK signaling linked to the schizophrenia-associated 1q21.1 deletion is required for neuronal and sleep maintenance
Source: PLoS Genet. 2018 Dec 19;14(12):e1007623. doi: 10.1371/journal.pgen.1007623 (PMC6317821; doi:10.1371/journal.pgen.1007623)

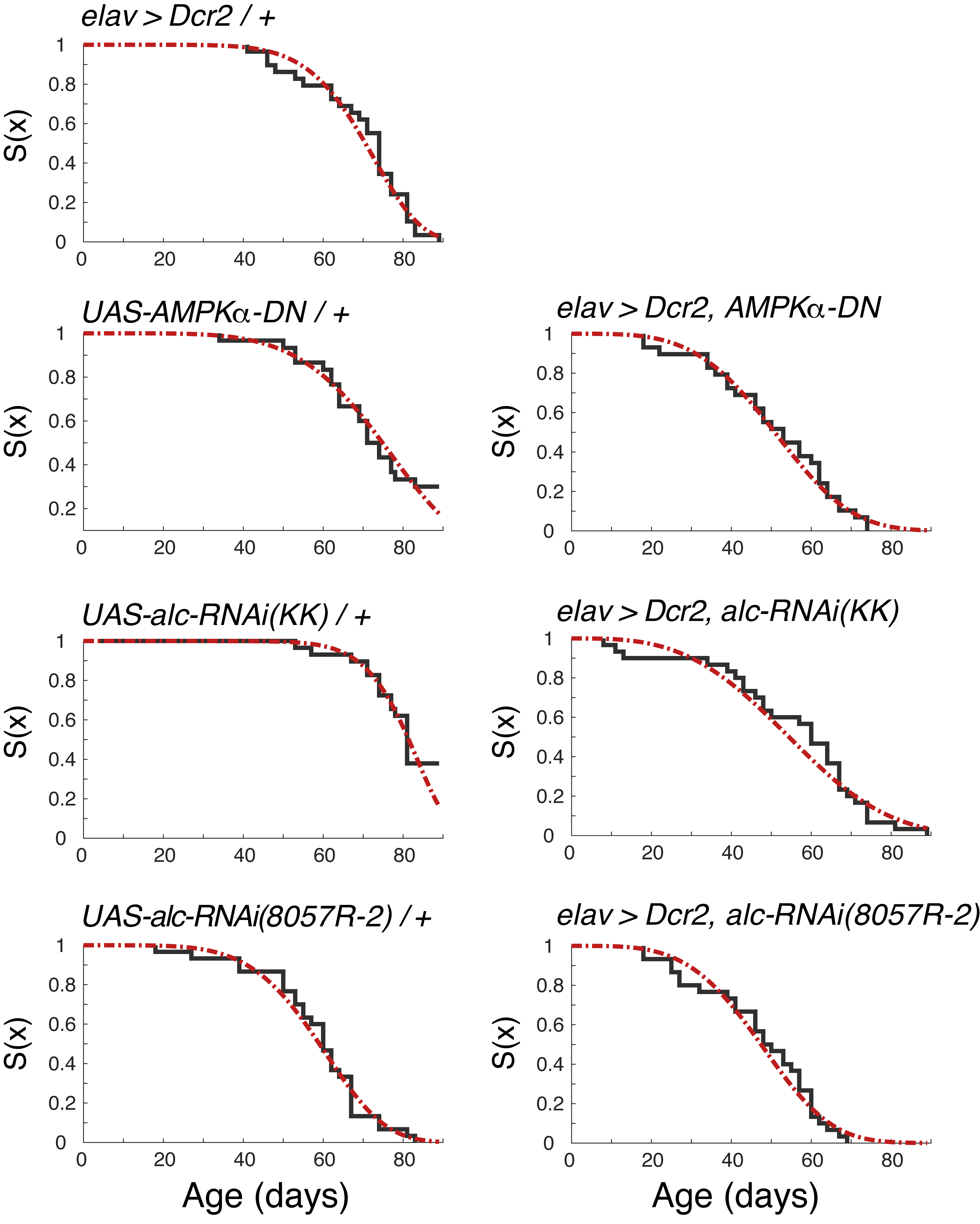

Supplement: S1 Fig — Example for all genotypes tested, showing fits of survival data, from individual vials of 30 animals, to a Weibull distribution. For controls, lines were crossed to w1118. (TIF) [file pgen.1007623.s001.tif]

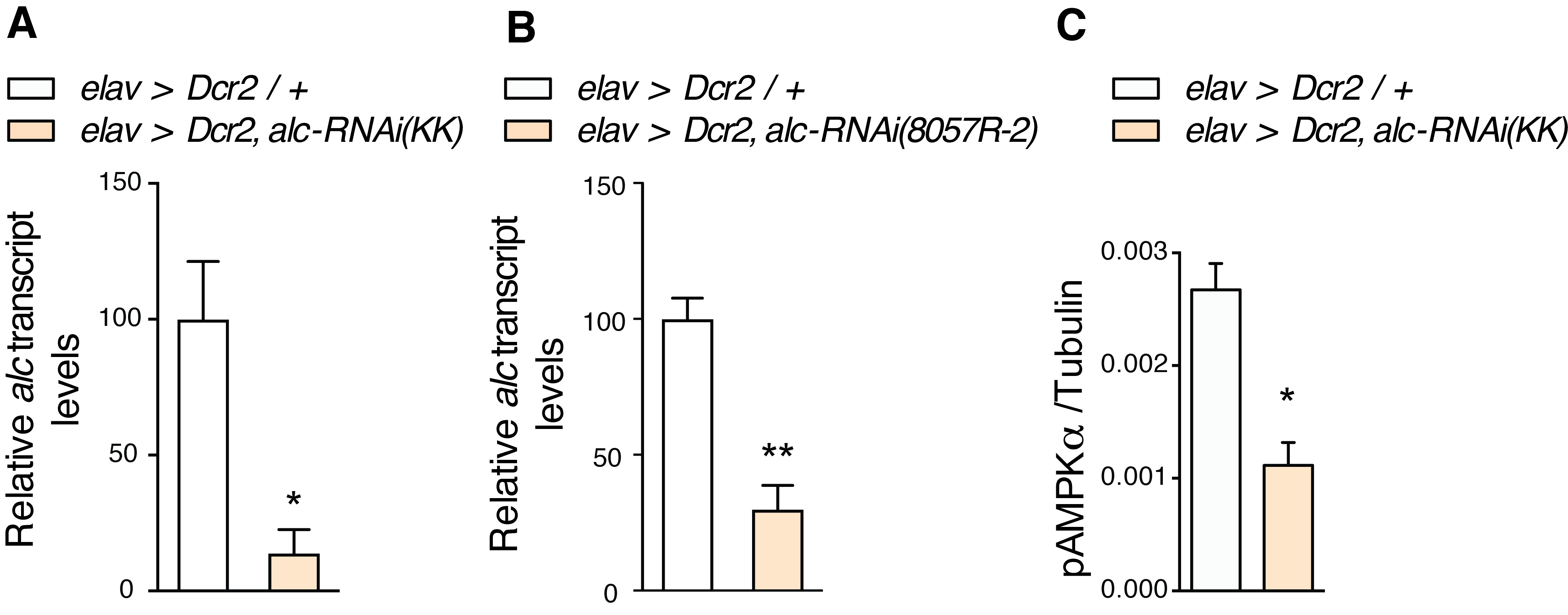

Supplement: S2 Fig — (A and B) Expression levels of alc mRNA in adult heads from animals with reduced neuronal expression of alc using two independent RNAi lines compared to controls. Data shown is relative to driver control. (C) Levels of pAMPKα are significantly reduced (-60% from controls) in adult heads when alc is knocked down pan-neuronally; α tubulin is used as a ratiometric loading control. Data from 4 experimental repeats using 8 adult heads per sample. For controls, lines were crossed to w1118. Error bars indicate SEM. Mann Whitney test was used to determine statistical significance: *p<0.05, **p<0.01, versus the control. (TIF) [file pgen.1007623.s002.tif]

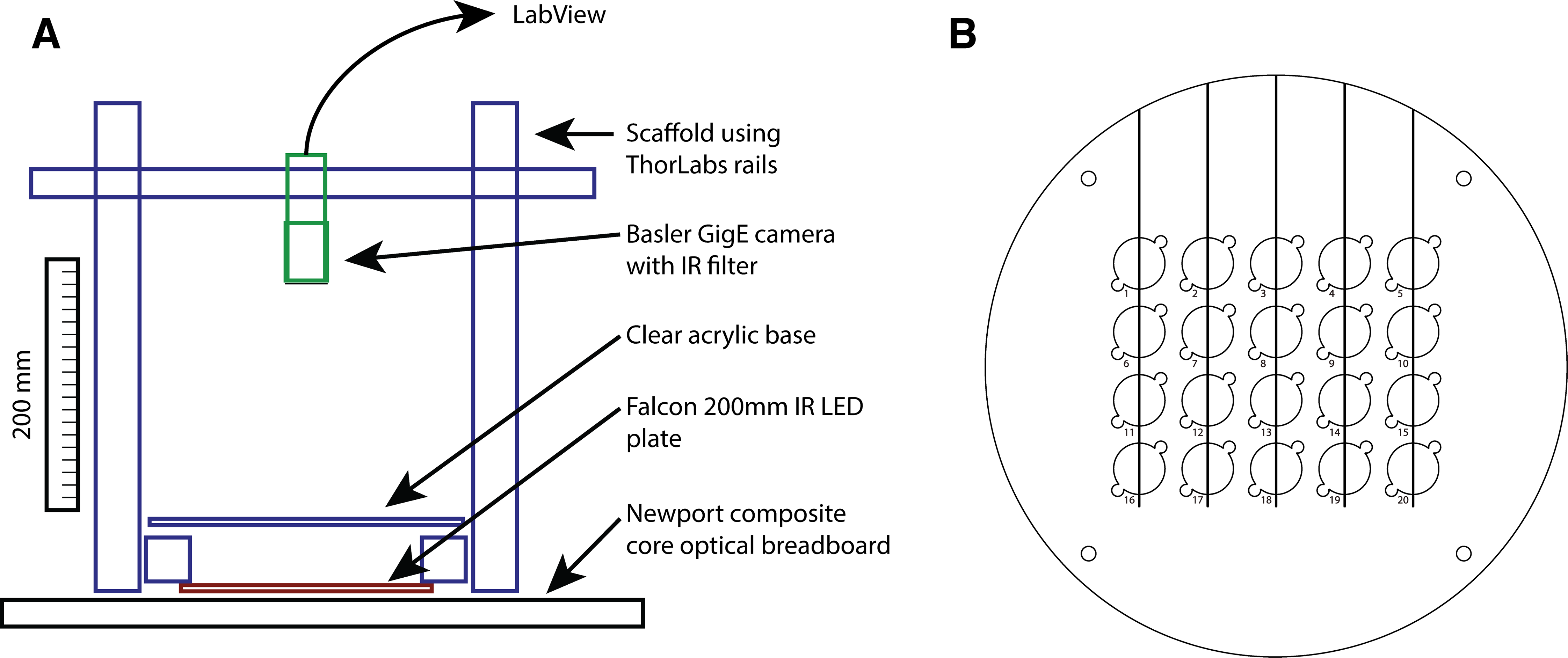

Supplement: S3 Fig — (A) Schematic diagram and components of the setup built to test courtship conditioning. (B) Schematic diagram of individual chambers laser-cut from acrylic that were used to test courtship conditioning. (TIF) [file pgen.1007623.s003.tif]

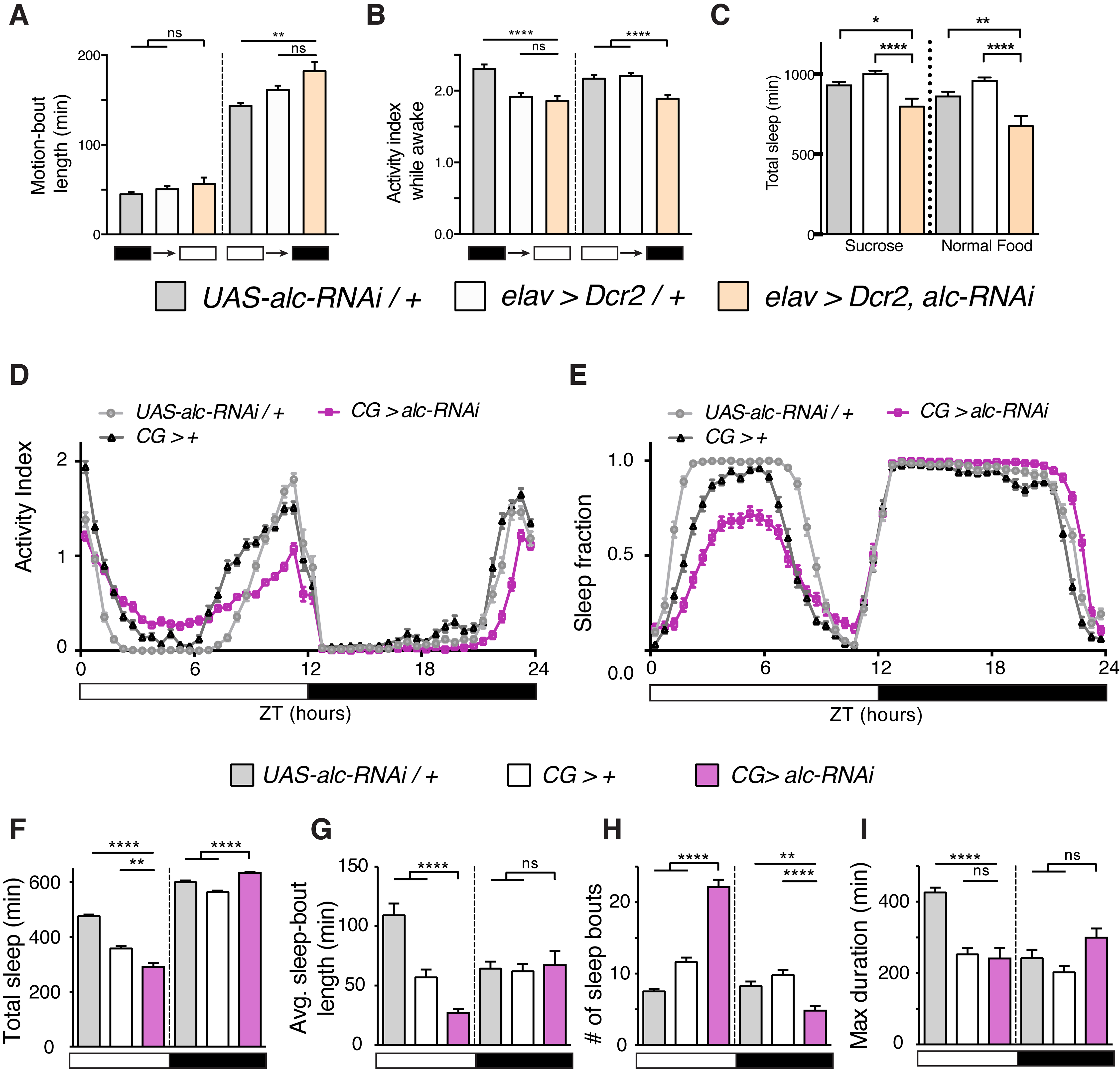

Supplement: S4 Fig — (A) Quantification of the average motion-bout length during dark-light and light-dark transitions (isolated as the motion bout present at t = 0(24) and t = 12 hours respectively) showed no significant differences in activity between elav>Dcr-2, alc-RNAi and control genotypes. (B) Quantification of activity (mean number of beam crossings per minute) during dark-light and light-dark transition periods showed a decrease in activity for elav>Dcr-2, alc-RNAi animals compared to control genotypes. (C) Total sleep over a 24-hour period for control genotypes elav>Dcr-2 and UAS-alc-RNAi/+ versus elav>Dcr-2, alc-RNAi on sucrose-based food and regular cornmeal food. For controls, lines were crossed to w1118. (D-E) Effects of fat body knockdown of alc. Activity (A) and sleep (B) profiles over a 24-hour period for control genotypes CG>+ (n = 32) and UAS-alc-RNA/+ (n = 32) versus CG>alc-RNAi (n = 32). All data obtained from second and third 24-hour cycle. Activity and sleep are shown in bins of 30 minutes. White and black bars represent ZT time, 12 hours light and 12 hours dark, respectively. (F) Total sleep (min) in CG>alc-RNAi flies with fat body alc knockdown shows reduced day time sleep and elevated night sleep compared to controls. (G) Average sleep-bout length (min) is significantly reduced during daytime in alc knockdown flies but not during night. (H) The number of sleep bouts per day increases when alc is knocked down in the fat body while being reduced during night sleep. (I) Maximum duration of sleep when alc is reduced in the fat body is unaffected except when comparing to UAS-alc-RNAi control during the day. For controls, lines were crossed to w1118. Error bars indicate SEM. Kruskal-Wallis test with Dunn’s post-hoc testing was used to determine statistical significance: *p<0.05, **p<0.01, ***p<0.001, ****p<0.0001, versus the control. (TIF) [file pgen.1007623.s004.tif]

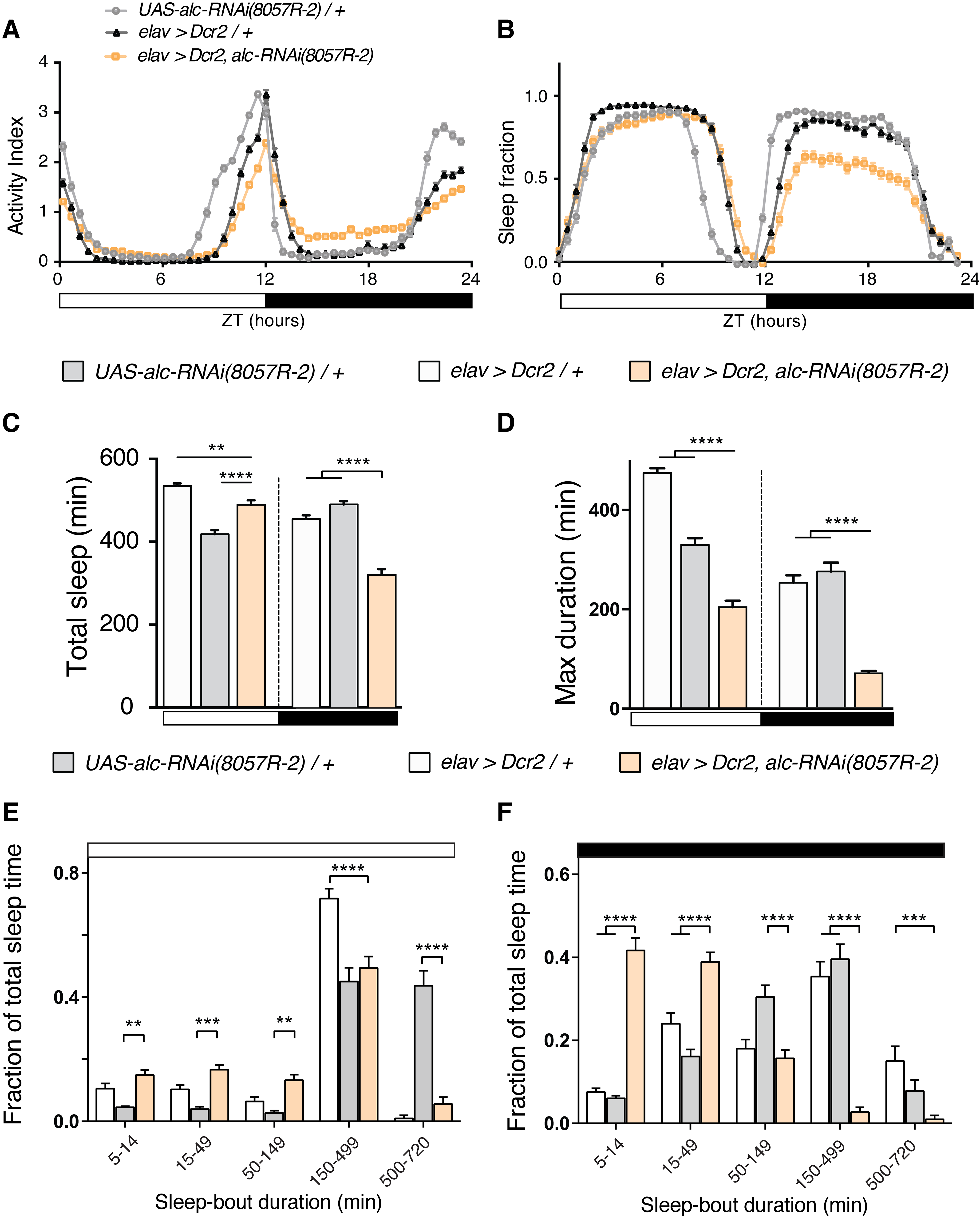

Supplement: S5 Fig — (A-B) Activity (A) and sleep (B) profiles over a 24-hour period for control genotypes elav>Dcr-2 (n = 32) and UAS-alc-RNAi(8057R-2)/+ (n = 32) versus elav>Dcr-2, alc-RNAi(8057R-2) (n = 32). All data obtained from second to fourth 24-hour cycle. Activity and sleep are shown in bins of 30 minutes. White and black bars represent ZT time, 12 hours light and 12 hours dark, respectively. (C) Total sleep (min) in flies with pan-neuronal alc knockdown compared to controls. Total sleep is significantly reduced compared to both controls during dark phase and to driver control in light phase when alc is knocked down in the nervous system. (D) Duration of the longest sleep bout (min) is significantly shorter in alc knockdown animals than in controls. (E, F) Distribution of length of sleep bouts for control genotypes elav>Dcr-2/+ and UAS-alc-RNAi(8057R-2)/+ versus elav>Dcr-2, alc-RNAi(8057R-2) during the day (E) and during the night (F). For controls, lines were crossed to w1118. Error bars indicate SEM. Kruskal-Wallis test with Dunn’s post-hoc testing was used to determine statistical significance: ***p<0.001, ****p<0.0001, versus the control. (TIF) [file pgen.1007623.s005.tif]

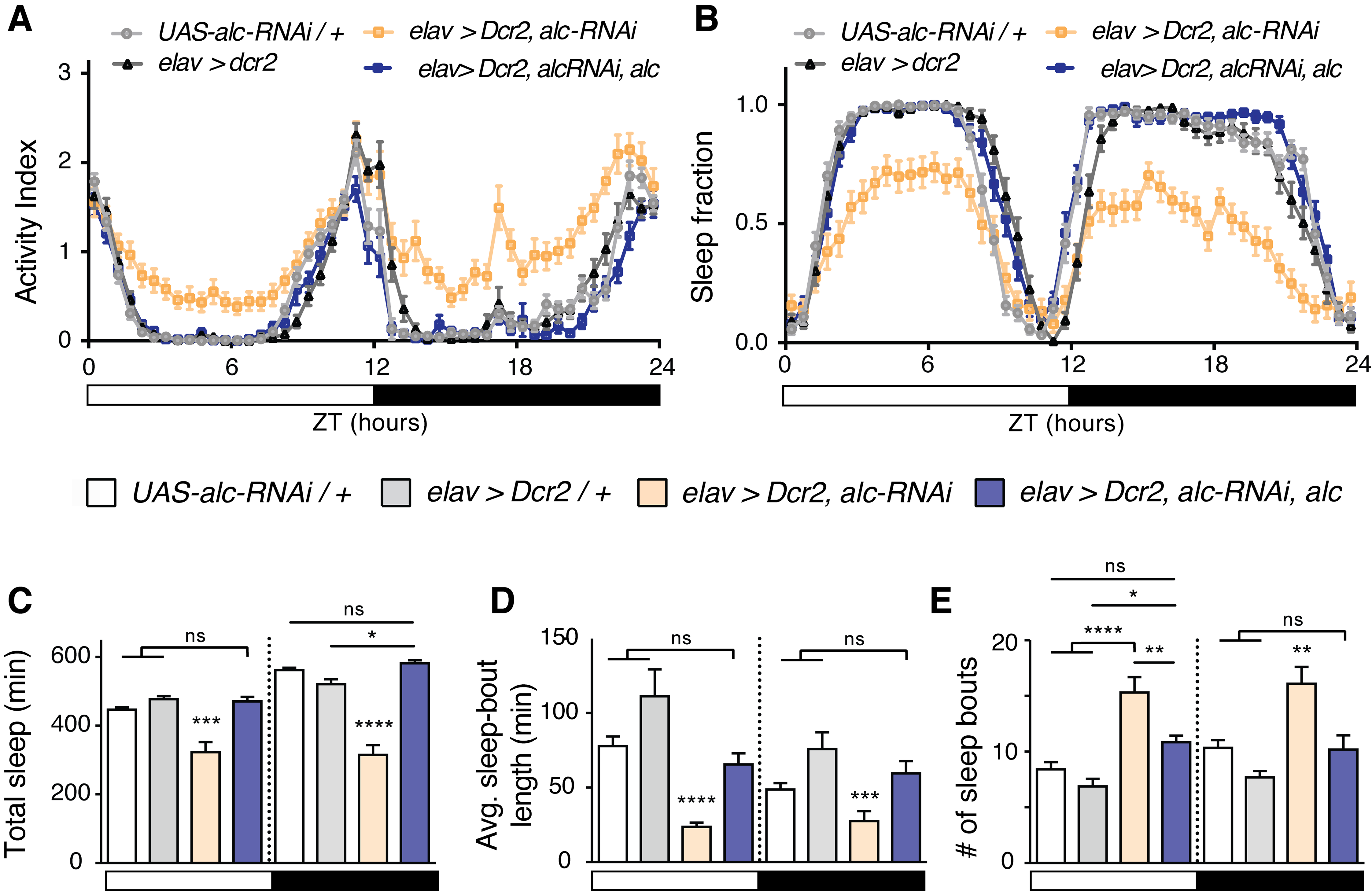

Supplement: S6 Fig — (A-B) Activity (A) and sleep (B) profiles over a 24-hour period for control genotypes elav>Dcr-2 (n = 16) and UAS-alc-RNAi/+ (n = 16) versus alc knockdown animals (elav>Dcr-2, alc-RNAi; n = 16) and alc overexpression animals (elav>Dcr-2, alc-RNAi, alc; n = 16). All data obtained from second and third 24-hour cycle. Activity and sleep are shown in bins of 30 minutes. White and black bars represent ZT time, 12 hours light and 12 hours dark, respectively. (C) Total sleep (min) in flies with pan-neuronal alc knockdown with and without alc overexpression compared to controls. (D) Average sleep-bout length (min) is significantly reduced during both day- and night-time in alc knockdown flies and rescued with alc overexpression. (E) The number of sleep bouts per day and night increases when alc is knocked down in the nervous system and rescued with alc overexpression. For controls, lines were crossed to w1118. Error bars indicate SEM. Kruskal-Wallis test with Dunn’s post-hoc testing was used to determine statistical significance: *p<0.05, **p<0.01, ***p<0.001, ****p<0.0001, versus the control. (TIF) [file pgen.1007623.s006.tif]

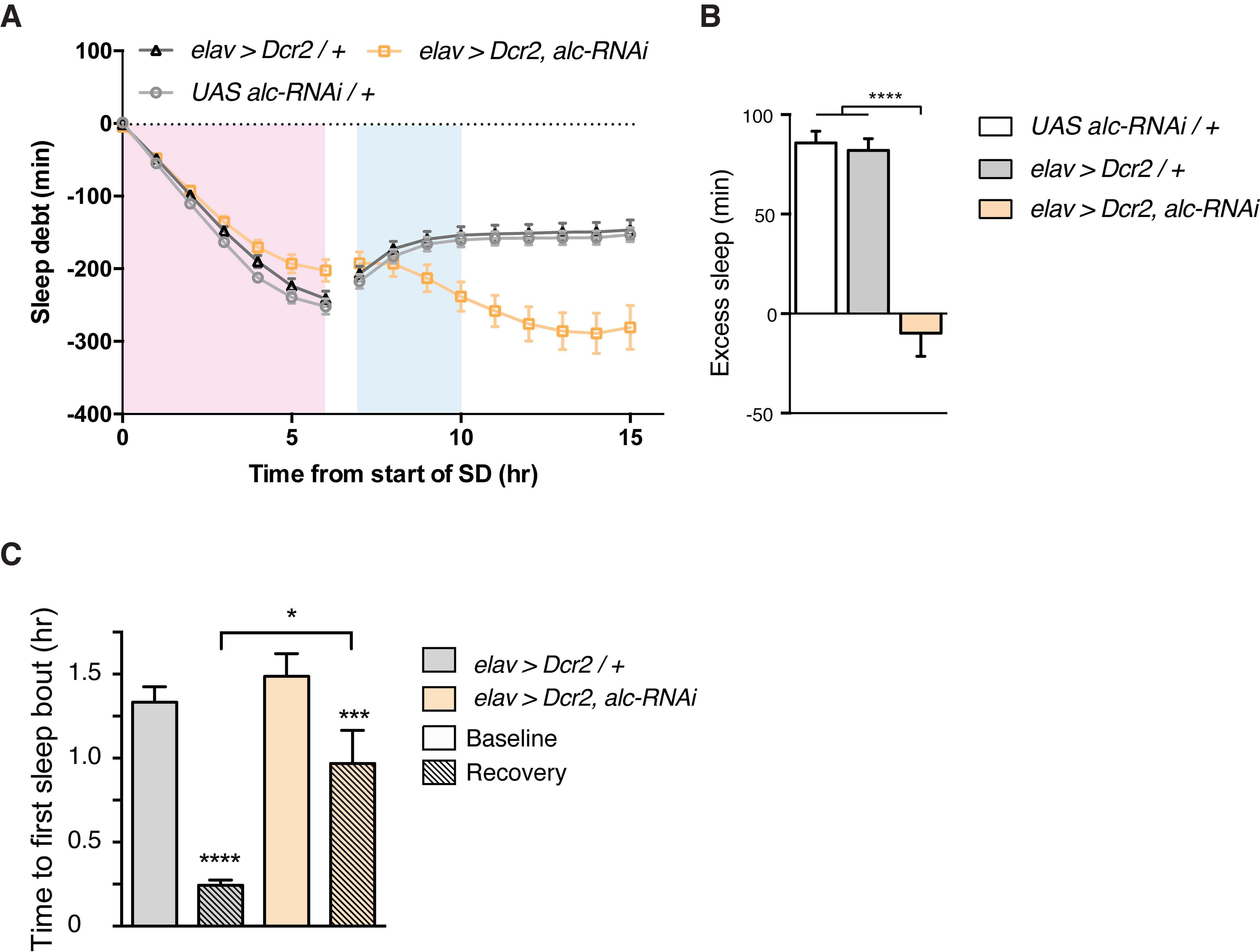

Supplement: S7 Fig — (A) Quantification of sleep debt (min) showing that both control elav>Dcr2/+ (n = 32) and UAS-alc-RNAi/+ (n = 32) flies recovered lost sleep in the first 3 hours following sleep deprivation while elav>Dcr-2, alc-RNAi(KK) (n = 32) animals with neuronal alc knockdown did not. (B) Both control genotypes showed a significant excess of sleep (~80 minutes) compared to the same time period of the baseline day while pan-neuronal alc-RNAi animals did not. (C) Time to first sleep bout (hours) is significantly reduced in both elav>Dcr2/+ (n = 54) and elav>Dcr2, alc-RNAi (n = 52) flies following sleep deprivation. For controls, lines were crossed to w1118. ****p<0.0001, versus the control. (TIF) [file pgen.1007623.s007.tif]

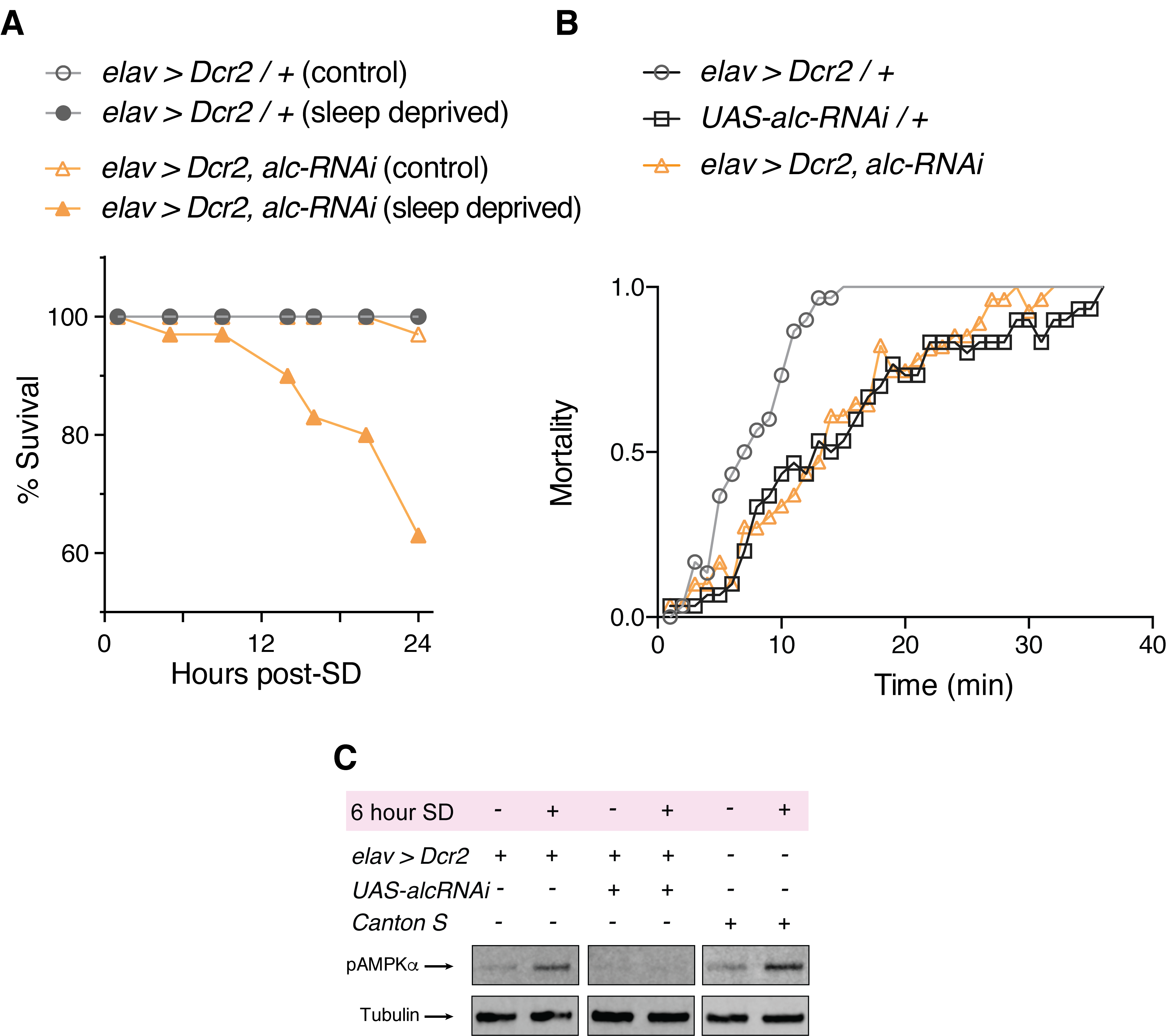

Supplement: S8 Fig — (A) Graph showing percentage survival and increased mortality of sleep deprived elav>Dcr2, alc-RNAi (n = 32) animals, compared to a sleep-deprived (SD) control genotype (elav>Dcr2/+, n = 32), and non-sleep deprived controls (elav>Dcr2/+, elav>Dcr2, alc-RNAi, n = 32). Survival was monitored over 24 hours post sleep deprivation, and the number of dead animals was counted. (B) Graph showing mean fraction mortality following a mechanical-stress assay (n = 3 vials of 10 animals for each genotype). Knockdown of alc in the nervous system does not increase susceptibility to mortality following mechanical stress. (C) Levels of phosphorylated AMPKα (pAMPKα) increased (normalized to alpha-Tubulin) immediately following 6 hours of sleep deprivation (SD) compared to same-time non-deprived animals for control animals (elav>Dcr-2/+ and Canton S). For controls, lines were crossed to w1118. (TIF) [file pgen.1007623.s008.tif]
